# Remote Work in a Changing World: A Nod to Personal Space, Self-Regulation and Other Health and Wellness Strategies

**DOI:** 10.3390/ijerph19084873

**Published:** 2022-04-17

**Authors:** Sybil Geldart

**Affiliations:** Psychology Program, Wilfrid Laurier University Brantford Campus, Brantford, ON N3T 2Y3, Canada; sgeldart@wlu.ca

**Keywords:** remote work, self-regulation, ergonomics, health and safety, management commitment

## Abstract

Remote work has escalated as a result of the coronavirus pandemic, and citizens have been doing their part to mitigate the spread of viral infection. The downside of quickly switching from a workplace office to remote work is that neither employees nor employers have had time to consciously process the new work environment and formally evaluate health and safety concerns. The aim of this commentary was to make suggestions on how to make remote work more satisfying, safe, and healthy for employees. First, I explored existing research on disease outbreaks and mental stress as the backdrop for discussing health-related strategies. To determine which types of strategies or measures would help, next I examined existing organizational research, including a qualitative study by my colleagues on workers’ perceptions about what makes a healthy workplace. Themes that emerged from the qualitative study align with three broad recommendations discussed in this commentary: cultivating personal space, building in ergonomics, and boosting self-regulation (self-learning) skills. Finally, I suggested that future research should explore the joint roles of the worker and his/her management team in recognition of organizational commitment to occupational health and safety alongside each worker’s need for autonomy in their personal workspace.

## 1. Introduction

An interest in occupational health and safety started 20 years ago after taking a post-doctoral position in the Epidemiology department at my alma mater. Trained as a psychologist I began to immerse myself in the work of my new supervisor, and together we investigated the role of organizational factors in workplace safety. Among many worthy findings what came out of the program of research was that management commitment was a significant correlate of lower injury rates, greater employee satisfaction and better mental health [1,2]. Managers who genuinely valued their employees, who spent money to improve working conditions and who promised to include subordinates in decision making regarding the use of personal protective equipment (PPEs) and other safety measures produced fewer workplace injuries and decreased reports of ill health. Our work took place over a decade ago and it is common knowledge today that, across organizations in developed and in developing countries, management commitment to occupational health and safety benefits everyone—workers themselves are found to be more satisfied and productive, and organizations reap accolades and higher profits for their noble deeds [3].

However, how, then, does health and safety get assessed when the occupation itself is happening outside of a physical workplace? Is there a gauge of management commitment when workers face hazards at a remote location? When it comes to work organization and its impact on health and safety, far less is discussed in the scientific literature regarding remote work conditions. Additionally, this is a problem because even before the coronavirus pandemic, variable work schedules and flexible locations have increased steadily, particularly in business, finance, and upper management occupations [4]. Remote work evolved over the last 20 years as it was valued by professionals who felt competent enough to work independently and who found themselves more productive when working outside the office [5], and because it lessened the pressure for busy working parents (mothers) to balance their time between a much-desired profession and home care responsibilities [6,7]. Indeed, remote work more than likely is here to stay. What is more, the need to maintain optimal health and safety when working from home is paramount given the digital world in which we live today and because of the trying times we continue to face as a result of a pandemic of this magnitude. 

The aim of this commentary is to make recommendations to both employers and employees on how to achieve occupational health and safety from the workers’ home. First, I draw upon existing research on disease outbreaks and mental stress to discuss challenges in performing work duties from home. This provides the context for discussing health-related strategies aimed to make remote work more satisfying, safe, and healthy for employees (and, in turn, more productive for organizations). To determine which strategies and measures would be particularly helpful within one’s own personal workspace, I refer to existing organizational research, including a 2008 study by MacDermid, Geldart, and colleagues [8] in which we interviewed female office workers, young retail workers, and male and female front-line workers and collected their views on work organization. We asked workers across diverse occupational groups to tell us what they envisioned as a healthy workplace and who they thought was responsible for creating a healthier workplace. A number of themes emerged from the data, including: the need for a safe environment, the need for support from supervisors and co-workers to mitigate work stress, enhanced communication between workers and upper management regarding safety procedures and policies, and better training and education of employees to keep up with advanced technology. These themes align with three broad recommendations discussed in the current paper and which arguably are in need of attention during changing and challenging times: cultivating personal space for work, building in ergonomics at the home office, and boosting self-regulation (self-learning) skills. First, let us look at some of the challenges of remote work.

## 2. Challenges of Work in a Changing World

Remote work has escalated since March 2020 across sectors as a result of the pandemic, with estimates for the U.S. workforce ranging from 35% to over 50% [9,10]. The good news is that citizens have been doing their part to mitigate the spread of viral infection—by removing themselves from social/public venues when possible. The bad news is that the switch to remote work was impromptu, and as such, employees and employers have had little time to process the changes in the work environment and evaluate health and safety issues stemming from remote work. The statistics on the prevalence of remote work today beg the question as to how organizations should be dealing with health and safety concerns of their workers who are now spending work time at a home base and less time in the office. 

What are some health and safety issues of remote work that ought to be addressed? We can make predictions about the adverse effects of COVID-19-related work changes by looking at previous research on disease outbreaks, as well as abundant research on overwork and work stress. SARS, Asian Avian Influenza A Virus (H5N1; 2014) and the Ebola virus (2014–2016) are disease outbreaks that were highly anxiety-provoking to the general public, especially in older adults [11,12]. Disease outbreaks put a special strain on the health care system. During the SARS outbreak in 2003, health care workers working in a Toronto teaching hospital reported feeling isolated, fatigued from understaffing and overwork, anxious about getting ill themselves, and conflicted about staying in their line of work in case they might transmit the disease to family members [13]. In hospital settings in Taiwan, health care workers felt conflicted by the desire to refuse work or terminate their job entirely, while those in quarantine felt stigmatized and rejected by neighborhood friends for being an essential worker and being exposed to SARS [14]. A recent systematic review and meta-analysis of studies examining the impact of COVID-19 in hospital settings reveals that psychological disorders related to anxiety and depression are highest in patients with a risk for viral infection yet are just as prevalent in health care workers as in the general public [15]. In organizations outside of health care, flu outbreaks have been known to put added stress on workers. Absences from sick employees means that employees who are left behind working—particularly those who fear losing their job and income—must deal with more job tasks than they can handle and longer work hours [16]. 

Living in a digital world means that employees across organizations have regular access to the internet at the workplace and can stay abreast of work outside of work hours thanks to smart phones and other personal devices. Faster internet at home makes computer work from home a viable option these days. Workers who stay digitally connected by phone or email respond to work tasks anytime it is convenient and can avoid travel time, traffic, and poor weather conditions [17]. However, work stress intensifies when there is a need to manage multiple responsibilities and maintain boundaries between work and home life.

Workers who have flexible work schedules or a flexible location of work value the time to manage family obligations. However, to ensure they stay on top of work matters remote workers typically report spending longer hours doing their job tasks from home [18]. They report the same degree of ‘work–family conflict’ [19] as workers who leave the household to go to the workplace office [20]. Workers with dependents have reported more stress in their day-to-day lives when schools closed temporarily to prevent the spread of the H1N1 virus [21]. Stress was reported also when parents took on the dual role of working at home while tutoring young children [22]. It is well known that when workload jeopardizes leisure time and interferes with caregiving and basic needs for personal growth and social connectedness, the result is higher reports of work dissatisfaction, depression, and burnout [23,24]. Adverse effects such as these would be expected given our longstanding coronavirus pandemic.

Will remote work be the new normal for businesses going forward? Across countries we have witnessed more than a few waves of infections. To this day, it is not clear whether new variants of coronavirus will cause another surge in infections and a need for the average working adult to switch out of the worksite office and back into homeworking. As a practicing psychologist in the province of Ontario, I took active steps to alter health care delivery and proceeded to see all my patients via Zoom, a video-conferencing platform. Telehealth is favored by therapists and turns out to be a common practice today among medical practitioners across Canada and elsewhere [25]. A quick shift like this towards remote work leads to important questions such as whether special skills, materials, and/or strategies will help professionals like myself conduct our jobs effectively going forward.

In the following section I will explore strategies and tips designed to help people in office work effectively and safely do their jobs from home. For each recommendation, it is important to consider the ways in which workers themselves play an integral role in achieving safety and health from a home base. It is, after all, their personal space and workers need to be self-aware of the constraints and limitations for doing work safely. However, workers are not alone in making improvements to work conditions even when work is being conducted in their own space. Workplace culture, vis-à-vis management attitudes and behaviors, must shift in a positive way in order to keep workers safe and healthy at their job—particularly when it is occurring from a novel location.

## 3. Recommendations for Working from Home

### 3.1. Cultivating Personal Workspace

It is necessary to cultivate personal space and some solitude when spending work time at home. That means turning off phones and other personal devices and moving the cell phone out of arm’s reach to avoid constant text messages and email correspondences. These are distractors that increase reaction time, lower attention to one specific task, and have the potential to reduce task performance and work productivity [26].

Distractions pose a problem for productivity, but so can a poor working environment. A theme that emerged from our previous qualitative study was the need for a safe environment [8]. One of the biggest complaints of office workers was having to perform with inadequate space, poor lighting, and poor ventilation. They believed that management failed to deal with the work environment. Therefore, what does this mean for office workers who are working from home? When working from home, workers are already familiar with the environment and will need to take the lead in assessing workplace concerns. A special room in the home dedicated for work may not be feasible for everyone; it will depend on the size of the home and the need to accommodate family members. Nor is dedicated workspace at home necessarily negotiated between family members ahead of time, which makes it challenging to achieve for some people [20]. However, privacy is helpful in order to create boundaries between work tasks and those activities and people that we associate with in home life [27]. A separate room or quiet location away from spouses, children, and family pets is advantageous for those who have poor attention and are easily distracted. Being watchful of loud conversations, the sound from TV, general noise levels, and air temperature are all important as well because extreme values have been known to lessen workers’ productivity and produce job dissatisfaction [28,29].

While we are on the topic of creating more solitude, let us not forget our current reality of unwavering distancing and repeated lockdowns. Any teachings about carving out more alone time likely would be met with some resistance today. In fact, the very thought of solitude may make some people feel more isolated and depressed. Another theme that emerged from our qualitative study of workers was the need for support. The results of our previous qualitative study revealed that positive interactions and humor among co-workers, along with social support from work peers, were seen as being vital to workers’ mental health [8]. Therefore, aloneness while working should not be too frequent and should not take up the majority of the day. I have advised my own clients, time and time again, that breaks are important to recharge and stay emotionally connected with friends and significant others. Break time is the perfect time to check social media and stay connected with other people. In other research, it has been suggested that employers be mindful of the potential for workplace loneliness and find ways to address employee well-being (e.g., via virtual lunch meetings; see Kniffin et al., 2021, for a discussion) [30]. Another way for organizations to help employees is to provide literature about working from home, to make regular contact and give feedback using video calls, and to ensure that employees have access to employee assistance programs and referrals to counselling services if needed [30].

Office workers with a significant amount of their workday doing computer work should be wary about how much of their actual day—including work and leisure—is consumed by personal devices and social media. Workers must take the initiative and assess for themselves whether or not they are over-using the internet at the cost of healthy behaviors. At the same time, employers should remind everyone of the need to take breaks throughout the workday and encourage physical activity and mind breaks. Employers of computer workers who are doing their work from home are advised to ask their employees ‘big-level questions’ such as “what do you need to limit distractions at home?” [31].

### 3.2. Building in Ergonomics

Musculoskeletal pain and injuries in computer users have been known to be correlated with poor posture, sitting for long periods, and the consistent use of inferior office equipment [32,33]. It has led companies to invest millions of dollars on ergonomic office equipment and devices. Ergonomic chairs were introduced into workplace offices as a way to support the upper limb and neck and prevent back injuries in office workers [34]. Special keyboarding devices have been designed to support the wrists and arms and prevent carpal tunnel syndrome [35]. Sit–stand desks are fairly new pieces of office equipment; their aim is to adjust posture and provide better circulation of the blood to body tissues [36], burn more calories, and presumably maintain a higher energy level. Engaging in daily regular exercise is another way to reduce musculoskeletal pain, enhance a person’s mood, and lower anxiety [37,38].

A significant amount of attention has been paid to improving office spaces and promoting physical activity at the workplace. It is for this reason that participants in our qualitative study remarked that ergonomics was not really a problem for them [8]. Office workers reported that their employers did a pretty good job of addressing problems and providing ergonomic interventions. However, what is still lacking is an assessment of the physical work conditions at workers’ own homes. Employers do not generally ask about the setup of workstations that belong to their employees. Employees may not even realize the potential for health problems due to poorly designed equipment and sedentary behaviors. Nor do they necessarily know that musculoskeletal disorders arise gradually over time and are difficult to correct if left untreated over the long term [39]. Frequent computer users must be vigilant about home office ergonomics. For intense computer and paperwork, best practices must be shared by organizations to enable workers to evaluate whether they are maintaining ergonomics at the home office. Workers should be instructed about what not to do, or what to avoid, when working from home. Are they doing computer work on the bed, at the kitchen table, or by sitting propped up against a living room sofa? Leaning against a headboard or sitting on the floor in front of the laptop can put the head in a forward tilt and put strain on the neck and upper shoulder. The computer monitor should be placed only two feet from the face, and the top of the screen should be placed at eye level. In fact, an ergonomic work chair and adjustable study desk, at minimum, provides an ideal height between the computer monitor and the viewer’s eye level. 

In a Birmingham Hospital Saturday Fund (BHSF) survey conducted with over 900 UK remote employees, it was noted that well over half the sample imagined their employer would not necessarily be interested in evaluating the ergonomics of their workspace at home [40]. I take this to mean that their employers did not take the time to openly speak to their subordinates about an ideal work environment and the need for proper equipment at home. Because of this, many of the employees who filled out the survey probably just assumed there was no commitment by management in improving health and safety outside of the workplace. As advised by Hall (2019), organizations that plan to offer homeworking should be prepared to conduct an assessment of the needs of the employee and share their occupational health concerns with home workers [40]. I would add that companies that enforce stay-at-home orders should be willing to invest the time and funds to ensure a safe and healthy working space at home. In turn, employees finding themselves working from home need to take a proactive approach by requesting that the employer evaluate their personal workspaces. Workers need to be encouraged to be good self-advocates by asking their employer directly to provide whatever materials and resources are necessary to improve a home working environment.

### 3.3. Boosting Self-Regulation Skills

Advances in technology, the rise in remote work in professional occupations and businesses, and the sudden change to working from home across organizations as a result of the coronavirus pandemic all have made it necessary for many employees to learn new methods and procedures for working at home. For me personally, I have had to eliminate face-to-face therapy sessions with clients and learn to use video conferencing as a way to deliver health care. I adapted my mode of therapy on short notice, but even today, I continue to see clients remotely from home. Telehealth was recommended by my provincial College at the start of the pandemic and has led to some rather significant changes in clinic procedures and policies around patient confidentiality and medical record keeping. As you can imagine, this drastic change in the way health care providers deliver services requires a fair bit of self-directed learning. 

Self-directed learning is necessary to use new software or resolve a task that is different from what one is accustomed to in the workplace. Self-directed learning relies on being an active participant [41], yet can be challenging when there is a tendency for poor attention and focus, and a tendency to be distracted easily by irrelevant stimuli in the learning environment [42]. Much of what we know about self-directed learning comes from research in education. In their review of studies examining the effectiveness of remote learning in students, Barbour and Reeves (2009) have noted that good results came from schools with students who took the initiative in doing schoolwork, who were motivated to excel, who monitored their performance, and who had fairly good time management skills among other strengths [43]. These are the very characteristics that make up self-learning and are found in self-regulated learners. 

As defined by Barry Zimmerman (2008), self-regulation is the ability to control thoughts and actions to achieve personal goals and respond to environmental demands [44]. Self-regulation is important for learners just as it is for stay-at-home workers essentially because workers are responsible for independently organizing materials and activities, following through with goals, and staying on top of job tasks. Remote workers should be encouraged to critically examine their own ability to self-regulate and decide if there are features or characteristics that they can change or would be willing to change. At the same time, companies that offer remote work need to evaluate the extent to which a given employee is capable of following task instructions without assistance. Questions need to be asked about many aspects of self-regulation. Does the worker know how to track his own progress? Does the worker have resources available to complete tasks? To whom would she turn to for help—by emailing a supervisor, by calling a co-worker for help, or perhaps by doing a quick Google search for information? It is important to consider what motivates the worker, and what rewards might help to get tasks started and finished. Does the worker have stamina to stay on task for long periods? Would he be willing to take breaks in an effort to manage his workload?

Questions also need to be addressed regarding the setting of work goals. One way to improve work from home is for the worker to practice setting achievable goals, and to request that the employer assist with goal setting. Workers who set realistic goals will be able to compare their progress and modify their tasks in order to meet the goal [45]. It is important for workers to see for themselves that they are successful at setting goals and achieving them. Once they see that they can complete work, workers will be more willing to exert more effort—which helps them feel confident about their performance [46]. In one recent study, workers who perceived themselves as confident and capable, i.e., self-efficacious, in adapting to home-office work during the pandemic reported that they felt supported by upper management and that there was good communication with their superiors for setting realistic goals in their new work environment [47]. Enhanced communication between workers and upper management and better training of employees to keep up with advanced technology were themes raised in our interviews of office workers. Feeling supported from supervisors and co-workers, either in the form of social-emotional support (e.g., being civil, trustworthy) or instrumental support (e.g., by providing training or assistance), was another important factor in workers’ health and well-being [8].

In summary, self-regulation skills are vital for employees to adapt to new work environments such as home. However, workers are not alone in the learning process. A successful transition to a home office means that both employees and employers are learning what it takes to boost worker productivity and enhance occupational health and safety.

## 4. Conclusions

The coronavirus pandemic caused a dramatic change in the way work is being conducted today. Work-from-home escalated since the start of the pandemic to ensure that people self-isolated, though clearly it is not a feasible approach for all occupation types. Additionally, whether it happens in any given organization depends on whether the business was successful in withstanding disruptions and revenue loss. Rather than doing any remote work at all, a number of citizens struggled financially during the pandemic as a result of being laid off, being asked to go on an unpaid leave of absence, or because they had been offered fewer hours of work than desired. For those who were allowed to continue working, many have seen abrupt changes in terms of where they perform job tasks and how they accomplish tasks.

As of this writing, many jurisdictions are easing restrictions and allowing people to go back to the workplace, at least for a portion of their time—in what is considered our *new normal*. However, the reality is that no one is really sure whether future COVID-19 outbreaks might change (again) the way citizens and employees conduct their business. Besides, in recent years, many organizations have permitted greater overlap between home and work to improve productivity and job satisfaction. Given our changing world, I have argued that we must be vigilant in maintaining optimal health and safety when working from home. More discussion is needed on what happens in the environment of the worker when switching from the outside workplace to the personal, individualized home office.

In this commentary, I reviewed some research exploring work challenges caused by a virus outbreak such as COVID-19. I offered recommendations in three broad areas which I believe are in need of attention as we navigate a global health crisis and new forms of work organization: creating personal space for work, introducing ergonomics at the home office, and developing or enhancing self-regulation skills. Workers today are juggling a myriad of tasks and from what I hear about in my clinic, many adults are struggling with caregiving responsibilities in addition to work demands. As they transition from workplace to home office, workers may be missing much-needed materials and resources. Office workers suddenly being asked to do job tasks remotely could do with a good dose of self-regulation and learning skills. 

I believe that organizations need to respect their employees’ home life and be responsible for teaching subordinates how to achieve, or maintain, health and safety conditions at home—which is a sure sign of organizational commitment. At the same time, workers themselves need to be independent and resilient in the face of adversity and empowered and free to articulate their need for information, materials, and counselling. In our qualitative study that explored workers’ perceptions of what would make a healthier workplace, many participants envisioned worker empowerment as one important solution. It fits with a shared decision-making approach towards optimal health and safety. On the whole, future research is needed to explore the joint or complementary roles of the worker and his/her management team in enhancing at-home occupational health and safety.
